# The Institutional Worker

**Published:** 1918-06-22

**Authors:** 


THE
### INSTITUTIONAL WORKER
**Being a Special Supplement to "The Hospital"**

### OUR BUREAU OF INFORMATION.

### Rules for Correspondents.

1. Every letter must be accompanied by the coupon to be cut from he back cover (inside page) of THE HOSPITAL, current issue, and aust contain the name and address of the correspondent with pseu-onym for publication if desired. Replies by post cannot be given ave under exceptional circumstances at the Editor's discretion.

2. Letters from Approved Homes in reply to special needs pub-ished in the Bureau should state terms and full particulars, and e sent prepaid under cover to the Editor of the Bureau with name ritten across coupon for identification.

3. Proprietors of Homes which have not yet been entered on the List of Approved Homes, but have spare accommodation likely to suit special needs, are invited to write for an application form for registration. The fee for registration, which includes two announcements of the Home in the Bureau and other privileges, is 10s.

4. All communications to be addressed to the Editor of THE HOSPITAL, 28 Southampton Street, Strand, London, W.C. 2, and marked "Bureau of Information."

### A Register of Nurses' Ward Work.

### Answer to April Question.

**By L. G.**

**THE question was :**

**How should a book be ruled to give at a glance a record of nurses' different duties in male, female and children's wards, and time spent at classes, etc.? The hospital is a training-school, and nurses take day and night duty. There are three floors for male patients, two for female patients, and a children's block.**

NOTE.—The following suggestions for keeping the book may usefully be followed.

**Cover of Book**—Name of Hospital. Nurses' Register—May 1918 to.........

**Size of Book**—About 13 in. wide by 11 in. deep.

**Name** ........ **Training** ...... **Years** ....... **Entered** .. .. **Accepted** ...... **Left**......

| Ward | Grade | Date | | No. of Months | Sister's Report | Lectures and Examinations | No. Classes | Marks | | Examiner's Remarks |
|---|---|---|---|---|---|---|---|---|---|---|
| | | From | To | | | | | Max. | Gained | |
| | | | | | | Anatomy & Physiology | | | | |
| | | | | | | Hygiene ... ... ... | | | | |
| | | | | | | Surgery ... ... ... | | | | |
| | | | | | | Medicine ... ... | | | | |
| | | | | | | Nursing ... ... ... | | | | |
| | | | | | | Bandaging ... ... | | | | |
| | | | | | | Dispensing ... ... | | | | |
| | | | | | | Massage ... ... ... | | | | |
| | | | | | | Eye ... ... ... | | | | |
| | | | | | | Ear, Nose and Throat | | | | |
| | | | | | | Cookery ... ... ... | | | | |

**Summary of Training**

| Nature of Work | Time | Remarks |
|---|---|---|
| Surgical ... ... ... ... | | |
| Medical ... ... ... | | |
| Children's ... ... ... ... | | |
| Gynæcology ... ... | | |
| Theatre ... ... ... ... | | |
| Massage ... ... ... ... | | |
| X-Ray ... ... ... ... | | |
| Casualty ... ... ... ... | | |
| Out-Patients ... ... ... | | |
| District ... ... ... ... | | |

**Day Duty**—To be filled in with black ink.

**Night Duty**—To be filled in with red ink.

**Nature of Certificate**

**Matron's Report**

### The Efforts of a Sanatorium Matron at Egg Production.

IN March and April 1917 I made three sittings of eggs, which only gave me eighteen chickens; eleven of these were cockerels.

The chickens and fowls have a free run, and they are fed on scraps from the kitchen and wards. One pullet began to lay in September—exactly at five months old. It laid five eggs a week the first month, and it has laid all through the winter months.

The total expenditure for the two years has been £2 3s., viz. : 1917—three broody hens and three dozen eggs, £1 10s.; 1918—two dozen eggs for hatching, 13s.; food, *nil*; which has produced : — 780 eggs, value £13 0s. 7d.; 11 cockerels and 3 hens for table use, worth £3 13s. 6d. On hand :—5 pullets worth 12s. 6d. each, £3 2s. 6d.; 2 mother hens worth 10s. 6d. each, £1 1s.; 4 chickens at 2s. 6d. each, worth 10s.; 8 chickens, at 2s. each, worth 16s.—total £22 3s. 7d.

| | £ | s. | d. |
|---|---|---|---|
| Total value of eggs, pullets, etc. ... ... ... ... | 22 | 3 | 7 |
| Total expenditure ... ... | 2 | 3 | 0 |
| Net profit | £20 | 0 | 7 |

[We shall be glad to receive accounts from other matrons of the result of their efforts.—ED. THE INSTITUTIONAL WORKER.]

### JUNE QUESTIONS.

**1. Equipping an X-ray Department.**

Give a list of equipment for an X-Ray, Electrical, and Massage Department in a hospital of 180 beds.

**2. Restrainer for Diphtheria Case.**

Describe a simple, washable restrainer, suitable for helping to keep flat in bed a young child suffering from diphtheria. Such a patient, after the first week or so, does not feel ill, and it is almost impossible to keep a child flat in bed without some kind of restrainer.

### RULES.

The following rules must be observed:—

1. Contributions must be written on one side of the paper. Brevity and terseness are desirable features. The MSS. must bear the name and address of the sender and be accompanied by coupon to be cut from the back cover (inside page) of the current issue of THE HOSPITAL. A pseudonym must be chosen if the name is not to be published.

2. Contributions must be addressed to the Editor of THE HOSPITAL, Institutional Worker Supplement, 28 & 29 Southampton Street, Strand, London, W.C. 2, should reach him before the end of the current month, and be marked in the left-hand corner "Facts and Figures."

**A minimum payment of Five Shillings will be made for each published answer.**

### QUESTIONS INVITED.

IN connection with our Question Box, we offer every month five shillings each for the two best questions which are sent in for consideration. The questions may be on any institutional subject and concern any department, from the secretary's to the porter's, or the matron's to the domestic staff; the one essential is that they must be practical and deal with points that have a definite relation to hospital or institutional administration, upkeep, finance, or management. Points relating to artificers' work, housekeeping, the laundry, out-patients, and other practical matters will be welcome. It is hoped that thus, when difficult or doubtful points arise in the course of their work, institutional workers will be encouraged to put them in the form of a question and send them to the Editor, THE HOSPITAL Bureau of Information, 28 & 29 Southampton Street, Strand, W.C. 2, marked "Question Box."

The best questions will be published in due course and our readers will be given the opportunity of answering them. Thus all institutional workers may be able to cooperate with the view of helping the work and smoothing the difficulties of each other. In short, we want the Question Box of the *Institutional Worker* to be freely used by every worker habitually.

### ENQUIRIES AND ANSWERS.

### *THE SICK AND IN NEED.*

**Sanatoria for Children.**

WE suggest that you write to the secretaries of the following institutions for full particulars of admission; at the same time give full particulars of the case you are interested in: Children's Sanatorium, Holt, Norfolk; Salop Convalescent Home, Baschurch, Shrewsbury; and the Sanatorium for Children, Harpenden, Herts. The Royal West of England Sanatorium, Weston-super-Mare, is for men and women.—MRS. T.

**Nervous Disorders.**

WE do not undertake to give medical advice in these columns or to recommend doctors. We suggest that you consult your usual medical adviser, who will suggest a specialist. Should you not be in a position to pay a specialist's fee or for the needful treatment you might make application to the National Hospital for the Paralysed and Epileptic, Queen Square, Bloomsbury, or to the special department at your own hospital at Manchester—the Royal Infirmary.—A. G. F.

### *EMPLOYMENT AND TRAINING.*

**Massage Certificate.**

THERE is no other certificate of massage equal to or better than that of the Incorporated Society of Trained Masseuses in this country.—C. H. W.

**Training-School at Bath.**

YES; the Royal United Hospital, Bath, is a recognised training-school. The training is for three or four years; the fourth year will, at the discretion of the board, be spent either in hospital or as a staff nurse or on the private nursing staff. Candidates are accepted at the age of twenty-one. Instruction is given in massage by a trained masseuse. Cooking classes also are held. Send your application to the matron.—BELL.

**Paying Probationer.**

UNIVERSITY COLLEGE HOSPITAL receives paying probationers for three years' training. The fees are: £30 for the first year; second year, free; third year a salary of £18 is given. Pupil-midwives are also received.—ASHMORE.

### *THE HOUSEKEEPERS' DEPARTMENT.*

**Care of Wood Flooring.**

THE wood flooring in the wards has, no doubt, been ruined by the use of very hot water and strong soap in the cleaning. It is necessary from a sanitary point of view that the floors should be washed at intervals apart from the regular polishing, but care should be taken to use only moderately warm water and mild soap. As soon as the floor is thoroughly dried, have it waxed and polished.—M. H.

**Butter-cutting Machine.**

SOME of the large hospitals in America use an excellent device for cutting their butter into portions, which results in a very great saving in butter; at one large New York hospital between 5 and 6 lb. of butter is saved in one day in this way. The utility of such a machine in this country at the present time is obvious, more especially for the cutting of margarine, which as a rule breaks when being cut in the ordinary way with a knife, thus causing a good deal of waste. The outfit is about 16 to 18 inches square, white porcelain inside and out, and is heavily insulated—a small quantity of ice will keep the butter at a proper temperature. The mechanism of the cutter is entirely enclosed, with automatic action for cutting. The cutter uses up every speck of butter; it holds 9 lb. of tub butter at a time, which is quickly inserted and cut into squares. Perhaps some of our readers may know of any like machine being used in this country. The makers of the American machine are the Glidden Manufacturing Company, 55 Broadway, Beverley, Mass.—HOSPITAL HOUSEKEEPER.

### *MISCELLANEOUS.*

**Railway Concessions to Nurses.**

THE privilege you refer to only applies to nurses at institutions which accommodate military as well as civil patients. Application, which must be made through your matron, should be addressed to the County Director of the London Territorial Force Association, Duke of York's Headquarters, Chelsea, S.W. 3.—BETTY.

**Loss by Local Purchases.**

IT is not wise to give patronage to your local tradespeople at the expense of the hospital funds. A hospital is in a position to purchase at wholesale prices, and unless your local tradespeople are prepared to let you have supplies at a price that allows them reasonable commission, there seems to be no sound reason for continuing your account with them. While, of course, it is only reasonable to purchase from local tradespeople if it be possible, the fact that they subscribe to the hospital does not justify foolish waste of funds. —K. O. R.

**First-aid for Foot Troubles.**

AN excellent little handbook on "First Aid for Foot Troubles and Battalion Chiropody," by Ernest G. V. Runting, President of the Incorporated Society of Chiropodists, has recently been published by The Scientific Press, Ltd., 28 and 29 Southampton Street, Strand, London, W.C. 2. You will find the suggestions on conducting classes in battalion chiropody extremely useful. —CHIROPODIST.

